# Synergistic Improvement in Children with Cerebral Palsy Who Underwent Double-Course Human Wharton's Jelly Stem Cell Transplantation

**DOI:** 10.1155/2019/7481069

**Published:** 2019-09-17

**Authors:** Xiaojun Fu, Rongrong Hua, Xiaodong Wang, Peishen Wang, Long Yi, Aixue Yu, Jing Yang, Yan Li, Yihua An

**Affiliations:** ^1^Department of Functional Neurosurgery, The 3rd Medical Center, Chinese People's Liberation Army General Hospital, No. 69 Yongding Road, Hai Dian District, Beijing 100039, China; ^2^The Graduate School, Xuzhou Medical College, No. 209 Tongshan Road, Xuzhou, Jiangsu 221004, China

## Abstract

**Background:**

Our previous studies confirmed that human Wharton's Jelly stem cell (hWJSC) transplantation improved motor function in children with spastic cerebral palsy (CP). This study investigated the dose-effect relationship between the transplanted cell dosage and efficacy in CP children.

**Methods:**

CP children who received one- or two-course (four or eight times lumbar puncture, 4 or 8 × 10^7^ hWJSCs) cell therapy were recruited into this study. Assessments of motor function were performed according to scales for gross motor function measurement (GMFM) and fine motor function measurement (FMFM). The measurement data obtained in the two different groups were analyzed by *t*-test. Univariate repeated measures analysis of variance was used to compare the data obtained at baseline and 6 or 12 months posttransplantation and met the conditions for Mauchly's sphericity test.

**Results:**

The results for fifty-seven pediatric CP patients (including 35 male and 22 female patients) who completed follow-up showed that gross and fine motor functions improved after cell therapy. Interestingly, the GMFM and FMFM scores in patients who received one course of transplantation were significantly increased at 6 months after treatment. Moreover, another course of transplantation further improved gross and fine motor function in children. The scores for GMFM and FMFM were significantly higher at 6 months posttransplantation than at baseline and showed a linear upward trend. There was no gender difference in GMFM. Interestingly, there was a significant difference between male and female patients in the B and C dimensions of FMFM. These results reveal a gender-related susceptibility to stem cell therapy, especially for movement capability of the upper extremity joint and grasping ability. Similarly, in the group aged ≤3 years old, the improvement observed in dimension A (lying and rolling) of GMFM was nearly exponential and showed a quadratic trend. The results for FMFM were similar to those for GMFM. Moreover, the improvement in motor function was not age dependent.

**Conclusions:**

In this study, our data collectively reveal that CP children display sex- or age-dependent responses to hWJSC therapy; these results shed light on the clinical utility of this approach in specific populations.

## 1. Background

Cerebral palsy (CP) is a permanent dysfunction that affects motor behavior and posture, thereby causing impaired mobility. CP affects approximately 1.5 to 4.5 live births per one thousand [1–3] and is a huge burden to society around the world. CP is mainly categorized as spastic, athetoid, or mixed CP, with spastic CP accounting for approximately 70-80% of the affected cases [4–6]. Traditionally, the treatments for spastic CP patients address its symptoms and include physical and occupational therapy [7], but their effects remain unsatisfactory. Furthermore, studies have led to new technical methods to treat spastic CP [8], including electrical stimulation therapy [9], nucleus lesioning, and selective dorsal rhizotomy (SDR) [10–13]. In the last few decades, stem cell therapy has opened up the horizon for treating patients with central nervous system disorders [14–24]. The application of autologous or allogeneic stem cells has also been attempted in CP patients. Related studies performed in both China and abroad, including our previous studies, have demonstrated that stem cell therapy can improve motor function in CP patients [25, 26].

Mesenchymal Stromal Cells (MSCs) are cells of mesodermal origin, which could be isolated from bone marrow, dermal skin, Wharton's Jelly, and other tissues [27]. They were described as “that fibroblast-like plastic-adherent cells, regardless of the tissue from which they are isolated” by the International Society for Cellular Therapy (ISCT) in 2005 [28]. One of the promising therapies for nervous system injury in the last few decades is the application of MSCs for their self-renewal capacity and multiple differentiation potential. The previous study revealed that mesenchymal stromal cells can promote the restoration of motor function in children with CP [29]; however, the dose-effect curve for this treatment has not been evaluated. In this study, we aimed to analyze whether a superposition affect is achieved in CP patients who received more cell doses via a double course of human Wharton's Jelly stem cell (hWJSC) transplantation.

## 2. Methods

### 2.1. Inclusion and Exclusion Criteria

The patients' guardians provided signed informed consent before recruitment and were allowed to choose one of two group assignments. Finally, sixty patients were recruited and fifty-seven subjects had completed the follow-up including thirty and twenty-seven patients in the one- and double-course groups, respectively. The inclusion criteria were as follows: (1) one-month-old to 12-year-old children diagnosed with spastic CP by two neurologists; (2) a Gross Motor Function Classification System (GMFCS) score between levels IV and V; and (3) no interference due to other related treatments, such as rehabilitation, traditional Chinese medicine, or surgery, within the three months prior to enrollment and during treatment. The exclusion criteria were as follows: (1) history of severe allergic, tumor, or autoimmune disease or intractable seizures; (2) positive serology for HIV, HBV, HCV, and syphilis; and (3) hereditary metabolic diseases of the nervous system.

### 2.2. Isolation and Culture of hWJSCs

**Figure 1:**
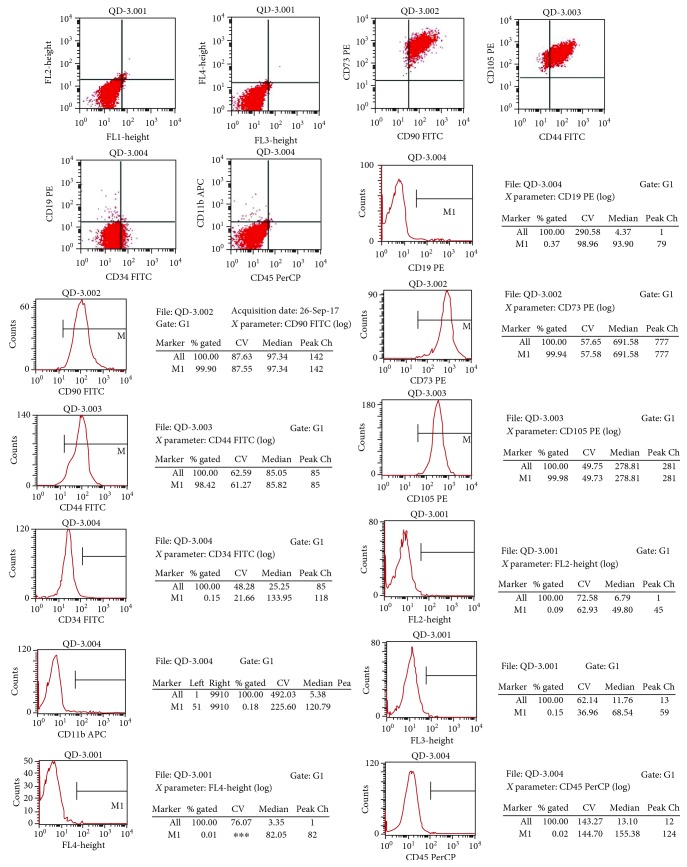
Cells were identified as hWJSCs by fluorescence-activated cell sorting. The percentages of cells expressing MSC markers (CD105, CD90, CD73, and CD44) were all higher than 95%, whereas those of cells expressing non-MSC markers (CD19, CD45, CD11b, and CD34) were no more than 0.1%.

Healthy pregnant women scheduled for cesarean section at full gestational age were selected and were asked to sign an informed consent form. An umbilical cord segment approximately 10 cm in length was removed after the baby was delivered. hWJSCs were isolated and cultured according to previously described methods [30]. Briefly, the cord was cut into cubes of approximately 0.5 cm^3^, which were washed with serum-free Dulbecco's modified Eagle's medium (DMEM) (Gibco Invitrogen, Carlsbad, CA, USA) and centrifuged at 250 g for 5 minutes. The cubes in the sediment component were cultured in the plate incubated with DMEM, 100 units/mL penicillin/streptomycin, and 10% FBS (STEMCELL Technologies Inc., Vancouver, BC, Canada) at 37°C for 10 days. After aspiration of the cord cubes, the adherent cells were trypsinized and recultured in a new flask. Cells were usually passaged every five to seven days and then stored in liquid nitrogen. Prior to transplantation, cells were thawed and passaged for three days. The hWJSCs harvested from passage 4 were used for a flow cytometry test. The percentages of mesenchymal stem cell (MSC) markers (CD105, CD90, CD73, and CD44) were all higher than 95%, whereas those of non-MSC markers (CD19, CD45, CD11b, and CD34) were no more than 1% (Figure 1). Finally, they were infused with normal saline by subarachnoid injection (2 mL).

### 2.3. Transplantation Protocol

After a routine examination excluded surgical contraindications, an intervertebral lumbar puncture was performed at L3-4 or L4-5 under local infiltration anesthesia. After confirming that the needle had entered the subarachnoid space, 1-2 mL of hWJSC suspension (containing 1 × 10^7^ stem cells) from the same donor was slowly injected into the subarachnoid space. Transplantation was conducted once every five to seven days, and one course was defined as four consecutive hWJSC transplantations via lumbar puncture. hWJSC retransplantations via lumbar puncture were performed after six months in CP children who accepted a double course of stem cell transplantation.

### 2.4. Efficacy Evaluation and Safety Assessment

The GMFM and FMFM scales were used to analyze changes in motor function pre- and posttransplantation. The GMFM scale is a popular assessment tool that includes five gross motor function domains and 88 items: A—lying and rolling (17 items, full score = 51), B—sitting (20 items, full score = 60), C—crawling and kneeling (14 items, full score = 42), D—standing (13 items, full score = 39), and E—walking, running, and jumping (24 items, full score = 72). On the FMFM scale, dimension A represents visual motion, B represents the movement capability of the upper extremity joint, C represents grasping ability, D represents operating capacity, and E represents hand-eye coordination.

### 2.5. Statistical Methods

SPSS 16.0 (SPSS Inc.; Cary, NC, USA) software was used for all statistical analyses. Charts were made using GraphPad Prism version 7 (GraphPad Software Inc.; San Diego, CA, USA). The measurement data obtained in the two different groups are expressed as the mean ± standard deviation and were analyzed by *t*-test. Univariate repeated measures analysis of variance was used to compare the data obtained at baseline and 6 or 12 months posttransplantation and met the conditions for Mauchly's sphericity test. *p* values < 0.05 were considered statistically significant.

## 3. Results

### 3.1. Changes in Motor Function in CP Patients Who Accepted One Course of Cell Therapy

**Figure 2:**
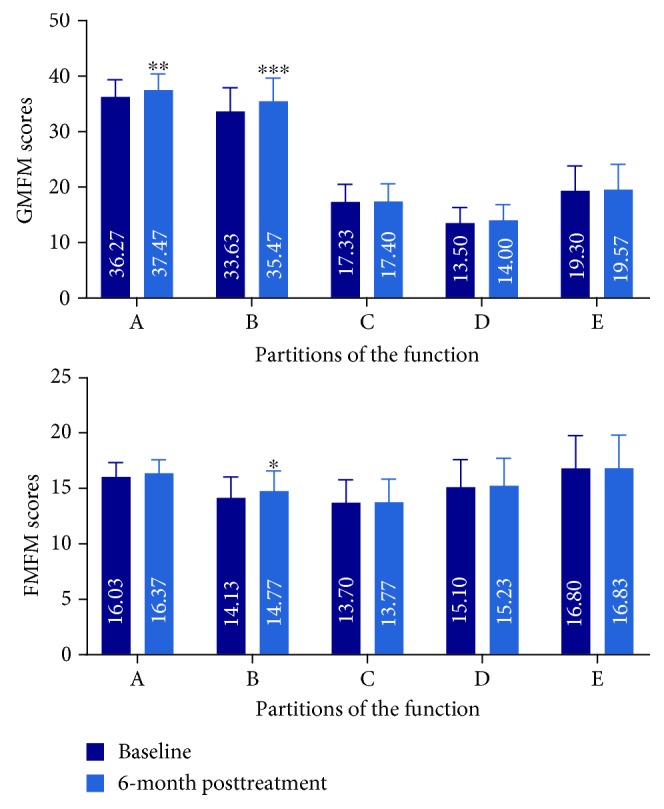
The GMFM and FMFM scores in CP patients treated with one course of hWJSCs. The GMFM and FMFM scores in CP patients who received one course of transplantation were significantly increased at 6 months after treatment. ^∗^*p* < 0.05, ^∗∗^*p* < 0.01, and ^∗∗∗^*p* < 0.001 compared with baseline. On the GMFM scale: A—lying and rolling (17 items, full score = 51), B—sitting (20 items, full score = 60), C—crawling and kneeling (14 items, full score = 42), D—standing (13 items, full score = 39), and E—walking, running, and jumping (24 items, full score = 72). On the FMFM scale, dimension A represents visual motion, dimension B represents the movement capability of the upper extremity joint, dimension C represents grasping ability, dimension D represents operating capacity, and dimension E represents hand-eye coordination.

Thirty CP patients were recruited into one course of cell therapy (group A), including 19 male and 11 female patients. The GMFM and FMFM scales were used to assess the motor function of the children with CP. In group A, CP patients accepted one course of cell therapy (four times lumbar puncture, 4 × 10^7^ hWJSCs). As shown in Figure 2, the scores for the GMFM A, B, C, D, and E dimensions were 36.27 ± 16.78, 33.63 ± 23.68, 17.33 ± 17.45, 13.50 ± 15.30, and 19.30 ± 24.64, respectively, resulting in a composite score of 119.37 ± 90.18 at baseline. After one course of cell therapy, the scores for the GMFM A, B, C, D, and E dimensions were 37.47 ± 15.94, 35.47 ± 22.79, 17.40 ± 17.45, 14.00 ± 15.57, and 19.57 ± 24.84, respectively, resulting in a total score of 123.20 ± 89.01, which was significantly higher than the pretreatment composite score (*p* < 0.001). At baseline, the scores for the FMFM A, B, C, D, and E dimensions were 16.03 ± 6.69, 14.13 ± 9.83, 13.70 ± 10.84, 15.10 ± 12.99, and 16.80 ± 15.40, respectively, resulting in a composite score of 75.77 ± 52.31. After one course of cell therapy, the scores for the FMFM A, B, C, D, and E dimensions were 16.37 ± 6.20, 14.77 ± 9.41, 13.77 ± 10.82, 15.23 ± 12.93, and 16.83 ± 15.43, respectively, resulting in a total score of 76.97 ± 51.32, which was significantly higher than the total score obtained pretreatment (*p* = 0.008).

### 3.2. Changes in Motor Function in CP Patients Who Accepted a Double Course of Cell Therapy

**Figure 3:**
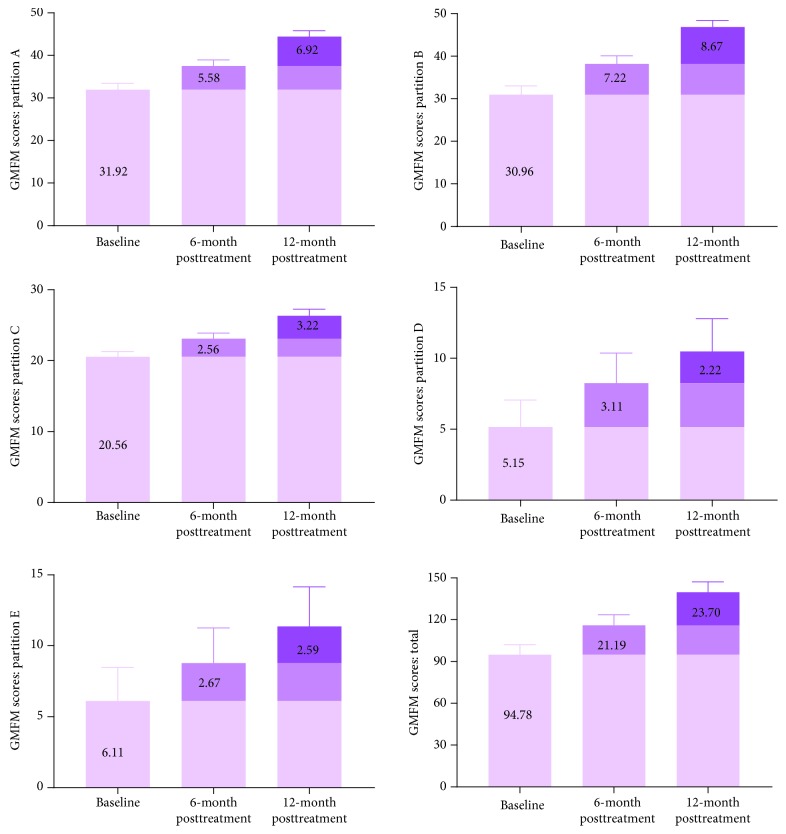
Increases of GMFM scores in CP patients who received two courses of hWJSC transplantation. The GMFM scores at 12 months posttransplantation were significantly higher than those at baseline or 6 months posttransplantation and showed a linear upward trend (*p* < 0.001, *p* < 0.001, *p* < 0.001, *p* = 0.015, and *p* = 0.001, respectively). On the GMFM scale: A—lying and rolling, B—sitting, C—crawling and kneeling, D—standing, and E—walking, running, and jumping. On the FMFM scale, dimension A represents visual motion, dimension B represents the movement capability of the upper extremity joint, dimension C represents grasping ability, dimension D represents operating capacity, and dimension E represents hand-eye coordination.

**Figure 4:**
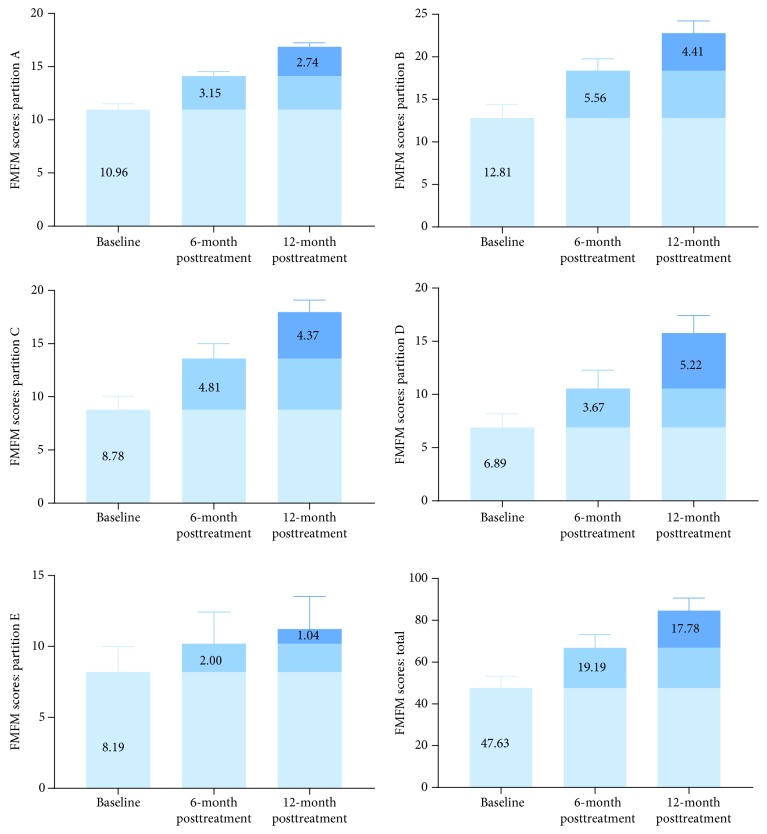
Increases of FMFM scores in CP patients who received two courses of hWJSC transplantation. The scores for FMFM were significantly higher at 12 months posttransplantation than at baseline or 6 months posttransplantation and showed a linear upward trend (*p* < 0.001, *p* < 0.001, *p* < 0.001, *p* < 0.001, and *p* < 0.001, respectively). On the GMFM scale: A—lying and rolling, B—sitting, C—crawling and kneeling, D—standing, and E—walking, running, and jumping. On the FMFM scale, dimension A represents visual motion, dimension B represents the movement capability of the upper extremity joint, dimension C represents grasping ability, dimension D represents operating capacity, and dimension E represents hand-eye coordination.

Twenty-seven CP patients were recruited into two courses of cell therapy (group B), including 16 male and 11 female patients. In group B, the CP patients accepted a double-course cell therapy (eight times lumbar puncture, 8 × 10^7^ hWJSCs). The results showed that the total scores for GMFM and FMFM were significantly higher at 6 months after one course of cell therapy. In addition, another course of cell transplantation further improved motor function in CP children. The delta values are shown in Figures 3 and 4. This result suggests that two courses of cell therapy produced a superposition effect on motor function in CP children.

### 3.3. Sex-Dependent Sensitivity to Stem Cell Treatment

The scores on the A, B, C, D, and E dimensions of the GMFM and FMFM were improved by cell therapy in CP patients despite a gender difference. At 12 months posttransplantation, the scores for the GMFM A, B, C, D, and E dimensions were 45.88 ± 5.49, 47.13 ± 8.40, 26.63 ± 4.38, 12.06 ± 13.00, and 13.13 ± 15.28, respectively, in male patients and 42.10 ± 9.17, 46.45 ± 7.84, 25.91 ± 5.50, 8.18 ± 10.67, and 8.82 ± 13.45, respectively, in female patients. All of these values were significantly higher than those obtained at baseline or 6 months posttransplantation and showed a linear upward trend (*p* < 0.001, *p* < 0.001, *p* < 0.001, *p* = 0.015, and *p* = 0.001, respectively). However, these differences disappeared when gender was added as a consideration (Table 1).

At 12 months posttransplantation, the scores on the FMFM A, B, C, D, and E dimensions were 16.69 ± 2.24, 21.94 ± 7.83, 17.94 ± 6.16, 16.00 ± 7.72, and 11.38 ± 11.44 in male patients and 17.09 ± 1.51, 24.00 ± 7.00, 18.00 ± 5.71, 15.45 ± 10.00, and 11.00 ± 13.18, respectively, in female patients. These values were significantly higher than those at baseline or 6 months posttransplantation, which formed a linear upward trend (*p* < 0.001, *p* < 0.001, *p* < 0.001, *p* < 0.001, and *p* < 0.001, respectively). Interestingly, there was still a significant difference in the B and C dimensions between male and female CP patients. The results revealed a gender susceptibility to stem cell therapy, especially regarding the B and C dimensions of FMFM (Table 2). The results in group A were similar (Table 3), providing further support for the finding that motor function was improved in CP children who accepted one course of cell therapy.

### 3.4. Effects of Age on Sensitivity to Stem Cell Treatment in CP Children

To explore the detailed mechanism underlying differences in sensitivity to cell therapy in CP patients, the subjects were divided into two groups according to their age. At 12 months posttransplantation, the scores on the GMFM A, B, C, D, and E dimensions were 43.28 ± 8.19, 46.18 ± 8.26, 26.13 ± 3.79, 10.75 ± 11.77, and 10.75 ± 13.90, respectively, in CP children aged ≤3 years old and 46.00 ± 5.57, 47.82 ± 7.96, 26.64 ± 6.14, 10.09 ± 13.00, and 12.27 ± 15.87, respectively, in CP children aged >3 years old. Hence, the scores were significantly higher at 12 months posttransplantation than at baseline and 6 months posttransplantation and showed a linear statistical trend (*p* < 0.001, *p* < 0.001, *p* < 0.001, *p* < 0.001, and *p* < 0.001, respectively). However, in the group aged ≤3 years old, the increases in dimension A of GMFM grew almost exponentially and showed a quadratic trend (Table 4). The results for FMFM were similar to those obtained for GMFM. Moreover, improvement in motor function was not age dependent (Table 5).

### 3.5. Adverse Reactions

1. Dizziness and headache: four patients in the experimental group and five patients in the control group showed low intracranial pressure within 48 h after surgery; symptoms included mild dizziness and headache and were occasionally accompanied by nausea and nonprojectile vomiting. The above symptoms were aggravated after ambulation and eased or eliminated when lying in the supine position. These patients were asked to remain in the supine position without pillows, and the symptoms were eased or eliminated after intravenous saline injection
2. Fever: two patients in the experimental group and four patients in the control group developed postoperative fever. All fevers were below 38.5°C and did not require treatment. The temperatures of these patients decreased to normal within 24 h. After the first course of treatment, six out of 57 children in the two groups suffered a slight fever (6/57); after the second course of stem cell therapy, only one out of 27 patients had a fever (1/27). Due to the limited number of samples, we could not confirm whether this difference was significant

## 4. Discussion

The current understanding of the mechanisms underlying MSC therapy is that either the transplanted MSCs home in on the targeted tissues, where they exert direct functions, or they modulate the systemic/local microenvironment without local implantation [31]. In nervous system diseases, stem cells can promote the generation of neuron-like cells and myelin-producing cells to reshape neural circuits. In addition, stem cells can transfer healthy MSC-derived mitochondria to neural stem cells (NSCs) and act in concert with endogenous neural stem cells [32–39]. In addition, the benefits of MSC administration are also attributable to its immunomodulation and paracrine effects. The results showed that MSCs may effectively suppress neuroinflammation, resulting in a reduction of the symptoms of neurological functional deficits by directly contacting immune cells or indirectly secreting cytokines, chemokines, and other soluble immunosuppressive factors [40–42].

Multiple reports have demonstrated that MSCs can promote the restoration of motor function in children with CP [29]. Ample evidence supports the notion that there are donor-dependent associations between directional differentiation capacity and vascular support of MSCs. Moreover, studies focused on the sexes of the donors have reported potential gender differences in the results of stem cell treatment of certain diseases [43–45], and these may be related to the expression of hormonal receptors [46, 47]. hWJSCs derived from Wharton's Jelly of the umbilical cord have been reported to possess the properties of both mesenchymal and embryonic stem cells [48]. In this study, the same origin-derived hWJSCs were used for a one- or two-course treatment with the aim of detecting different responses among individual recipients. The results showed that gross and fine motor functions improved in CP children who received hWJSC transplantation despite differences in the numbers of cells received. In particular, the scores for all dimensions of GMFM and FMFM were significantly higher in patients who accepted one course of cell therapy. Unexpectedly, performing a second course of cell therapy after a six-month interval increased the scores of all dimensions in the GMFM and FMFM scales. The double course of cell therapy could produce a superposition effect on motor function accessed by GMFM and FMFM scales. Hence, to improve gross and fine motor function, it may be worth trying two or more courses of stem cell transplantation in CP children.

Interestingly, we observed that CP patients exhibited sex- or age-dependent sensitivity to stem cell treatment. The scores for all dimensions in the GMFM and FMFM scales showed linear upward trends over time. Additionally, there was a significant difference in the B and C dimensions of FMFM between male and female CP patients. The improvement in the movement capability of the upper extremity joints and grasping ability were better in female patients than in male patients. These results support the notion of gender-related susceptibility to stem cell therapy, especially in the B and C dimensions of FMFM (in which B represents movement capability of the upper extremity joint and C represents grasping ability). This finding suggested that patients with repeated treatments or female gender will benefit more from hWJSC transplantation than other patients, especially with regard to the function of the upper extremity, although all gross and fine motor functions improved linearly. Of course, depending on developmental process, it has been speculated that improvements in lower limb function may require longer observation times, and this is one direction of our future research study. Similarly, while the scores for all dimensions of the GMFM and FMFM scales showed linear upward trends in CP patients across different age groups, the increases observed in the group aged ≤3 years old were almost exponential, showing a quadratic trend.

## 5. Conclusions

In this study, our data collectively reveal that CP children display sex- or age-dependent responses to hWJSC therapy; these results shed light on the clinical utility of this approach in specific populations.

## Figures and Tables

**Table 1:** The GMFM scores in double-course hWJSC-treated CP patients of different genders.

	Male (*n* = 16)			Female (*n* = 11)			*p* values for time	*p* values for time∗gender
	Baseline	6 months	12 months	Baseline	6 months	12 months		
A	33.88 ± 6.02	39.31 ± 5.44	45.88 ± 5.49	28.80 ± 9.35^∗^	34.60 ± 9.67	42.10 ± 9.17	<0.001 (linear)	>0.05
B	31.81 ± 10.34	38.75 ± 10.51	47.13 ± 8.40	29.73 ± 11.62	37.36 ± 9.92	46.45 ± 7.84	<0.001 (linear)	>0.05
C	21.63 ± 3.48	24.31 ± 3.70	26.63 ± 4.38	19.00 ± 3.69	21.36 ± 4.34	25.91 ± 5.50	<0.001 (linear)	>0.05
D	5.81 ± 10.63	9.63 ± 11.66	12.06 ± 13.00	4.18 ± 9.06	6.27 ± 10.12	8.18 ± 10.67	0.015 (linear)	>0.05
E	7.38 ± 13.99	11.06 ± 14.09	13.13 ± 15.28	4.27 ± 9.70	5.45 ± 10.69	8.82 ± 13.45	0.001 (linear)	>0.05
Total	100.50 ± 38.15	123.06 ± 39.98	144.81 ± 39.66	86.45 ± 37.03	105.64 ± 37.94	132.18 ± 38.41	<0.001 (linear)	>0.05

^∗^ *p* < 0.05, compared with the baseline in male patients.

**Table 2:** The FMFM scores in double-course hWJSC-treated CP patients of different genders.

	Male (*n* = 16)			Female (*n* = 11)			*p* values for time	*p* values for time∗gender
	Baseline	6 months	12 months	Baseline	6 months	12 months		
A	11.31 ± 3.18	14.38 ± 2.45	16.69 ± 2.24	10.45 ± 2.21	13.73 ± 1.79	17.09 ± 1.51	<0.001 (linear)	>0.05
B	13.63 ± 7.30	18.38 ± 7.16	21.94 ± 7.83	11.64 ± 9.56	18.36 ± 7.65	24.00 ± 7.00	<0.001 (linear)	0.004 (linear)
C	9.75 ± 5.63	14.06 ± 6.84	17.94 ± 6.16	7.36 ± 8.04	12.91 ± 8.48	18.00 ± 5.71	<0.001 (linear)	0.038 (linear)
D	6.50 ± 5.73	10.50 ± 8.91	16.00 ± 7.72	7.45 ± 8.09	10.64 ± 9.38	15.45 ± 10.00	<0.001 (linear)	>0.05
E	8.38 ± 9.39	10.06 ± 11.47	11.38 ± 11.44	7.91 ± 9.74	10.36 ± 12.31	11.00 ± 13.18	<0.001 (linear)	>0.05
Total	49.56 ± 26.86	67.38 ± 33.35	83.94 ± 31.42	44.82 ± 34.02	66.00 ± 35.02	85.55 ± 31.7	<0.001 (linear)	>0.05

**Table 3:** The GMFM scores in one-course hWJSC-treated CP patients of different genders.

	Male (*n* = 16)		*p* values	Female (*n* = 11)		*p* values
A	33.88 ± 6.02	39.31 ± 5.44	<0.001	28.80 ± 9.35	34.60 ± 9.67	<0.001
B	31.81 ± 10.34	38.75 ± 10.51	<0.001	29.73 ± 11.62	37.36 ± 9.92	<0.001
C	21.63 ± 3.48	24.31 ± 3.70	<0.001	19.00 ± 3.69	21.36 ± 4.34	<0.001
D	5.81 ± 10.63	9.63 ± 11.66	<0.001	4.18 ± 9.06	6.27 ± 10.12	<0.001
E	7.38 ± 13.99	11.06 ± 14.09	<0.001	4.27 ± 9.70	5.45 ± 10.69	<0.001
Total	100.50 ± 38.15	123.06 ± 39.98	<0.001	86.45 ± 37.03	105.64 ± 37.94	<0.001

**Table 4:** The GMFM scores in double-course hWJSC-treated CP patients of different ages.

	Age						*p* values	
	<3 years			3 years < age < 6 years			*p* values for time	*p* values for time∗age
	Baseline	6 months	12 months	Baseline	6 months	12 months		
A	29.93 ± 8.36	36.53 ± 8.71	43.28 ± 8.19	34.64 ± 6.09	38.82 ± 5.72	46.00 ± 5.57	<0.001 (linear)	0.031 (quadratic)
B	30.06 ± 11.39	37.56 ± 10.74	46.18 ± 8.26	32.27 ± 10.01	39.09 ± 9.53	47.82 ± 7.96	<0.001 (linear)	>0.05
C	19.81 ± 3.56	22.63 ± 3.96	26.13 ± 3.79	21.64 ± 3.88	23.82 ± 4.53	26.64 ± 6.14	<0.001 (linear)	>0.05
D	3.25 ± 7.84	7.31 ± 10.20	10.75 ± 11.77	7.91 ± 12.14	9.64 ± 12.41	10.09 ± 13.00	=0.001 (linear)	>0.05
E	5.13 ± 11.99	7.75 ± 12.79	10.75 ± 13.90	7.55 ± 13.22	10.27 ± 13.53	12.27 ± 15.87	<0.001 (linear)	>0.05
Total	88.44 ± 35.66	112.06 ± 39.22	137.50 ± 38.16	104.00 ± 40.22	121.64 ± 40.84	142.82 ± 41.66	<0.001 (linear)	>0.05

**Table 5:** The FMFM scores in double-course hWJSC-treated CP patients of different ages.

	Age						*p* values	
	<3 years			3 years < age < 6 years			*p* values for time	*p* values for time∗age group
	Baseline	6 months	12 months	Baseline	6 months	12 months		
A	11.25 ± 3.13	14.56 ± 2.00	17.50 ± 1.71	10.55 ± 2.34	13.45 ± 2.38	15.91 ± 1.97	<0.001 (linear)	>0.05
B	12.19 ± 7.50	19.00 ± 6.36	23.81 ± 6.72	13.73 ± 9.38	17.45 ± 8.56	21.27 ± 8.47	<0.001 (linear)	>0.05
C	8.00 ± 6.88	13.00 ± 6.73	18.06 ± 5.25	9.91 ± 6.52	14.45 ± 8.57	17.82 ± 6.94	<0.001 (linear)	>0.05
D	6.31 ± 7.12	9.69 ± 8.24	14.81 ± 7.93	7.73 ± 6.15	11.82 ± 10.12	17.18 ± 9.57	<0.001 (linear)	>0.05
E	6.44 ± 7.80	8.25 ± 9.67	9.00 ± 10.48	10.73 ± 11.15	13.00 ± 13.92	14.45 ± 13.64	<0.001 (linear)	>0.05
Total	44.19 ± 29.04	64.50 ± 29.78	83.19 ± 28.65	52.64 ± 30.70	70.18 ± 39.30	86.64 ± 37.08	<0.001 (linear)	>0.05

## Data Availability

The data that support the findings of this study are available upon request from the corresponding author. The data are not publicly available due to privacy concerns or ethical reasons.

## Abbreviations

hWJSCs: Human Wharton's Jelly stem cells
CP: Cerebral palsy
MSCs: Mesenchymal stromal cells
FDA: Food and Drug Administration
GMFCS: Gross motor function classification system
GMFM: Gross motor function measurement
FMFM: Fine motor function measurement.

## Additional Points

*Highlights*. (1) CP children display sex- or age-dependent responses to hWJSC therapy. (2) Synergistic improvement was observed in CP children who underwent double-course hWJSC transplantation.

## Supplementary Materials

Supplementary Materials Supplementary Table 1: GMFM scores of patients who accepted double-course hWJSC transplantation. Supplementary Table 2: FMFM scores of patients who accepted double-course hWJSC transplantation. Supplementary Table 3: GMFM scores of patients who accepted one-course hWJSC transplantation. Supplementary Table 4: FMFM scores of patients who accepted one-course hWJSC transplantation.
