# Supplementary material for: Synergistic Improvement in Children with Cerebral Palsy Who Underwent Double-Course Human Wharton's Jelly Stem Cell Transplantation
Source: Stem Cells Int. 2019 Sep 17;2019:7481069. doi: 10.1155/2019/7481069 (PMC6766101; doi:10.1155/2019/7481069)
Supplement: Supplementary Materials — Supplementary Table 1: GMFM scores of patients who accepted double-course hWJSC transplantation. Supplementary Table 2: FMFM scores of patients who accepted double-course hWJSC transplantation. Supplementary Table 3: GMFM scores of patients who accepted one-course hWJSC transplantation. Supplementary Table 4: FMFM scores of patients who accepted one-course hWJSC transplantation. [file 7481069.f1.pdf]

Supplementary table 1. GMFM scores of patients accepted double-course hWJSCs transplantation

			Baseline						6-month after treatment						12-month after treatment					
NO.	gender	age	A	B	C	D	E	Total	A	B	C	D	E	Total	A	B	C	D	E	Total
1	F	36	34	30	15	1	0	80	43	40	18	2	0	103	48	50	23	2	0	123
2	F	36	30	23	14	1	0	68	36	33	17	2	0	88	49	41	20	2	0	112
3	F	7	31	26	19	0	0	76	38	38	20	1	0	97	49	48	22	17	22	158
4	M	24	42	27	23	1	0	93	46	39	25	1	2	113	48	50	28	3	2	131
5	M	36	27	18	21	0	0	66	34	24	28	0	0	86	45	37	30	1	1	114
6	F	96	40	45	28	29	32	174	45	50	33	31	34	193	49	56	40	31	40	216
7	F	36	17	19	20	0	0	56	22	27	22	0	1	72	32	35	29	0	1	97
8	F	10	18	19	19	0	0	56	23	27	22	0	0	72	29	38	27	1	0	95
9	M	60	41	49	21	29	33	173	45	53	24	29	34	185	50	60	27	33	40	210
10	F	24	18	19	20	0	0	57	23	26	23	1	0	73	28	39	27	1	0	95
11	M	72	35	21	20	1	0	77	40	27	21	3	1	92	50	46	26	3	1	126
12	M	48	18	22	20	0	0	60	27	29	23	0	0	79	33	41	27	1	1	103
13	M	96	35	46	28	21	16	146	40	55	30	23	19	167	49	60	31	23	19	182
14	F	36	#NULL!	36	16	1	6	93	41	40	18	17	13	129	50	46	23	17	16	152
15	M	24	35	35	20	1	0	91	43	43	23	7	13	129	48	51	24	27	21	171
16	M	36	33	40	18	4	5	100	41	49	21	13	12	136	49	55	23	20	16	163
17	M	132	33	35	17	6	2	93	36	40	19	14	14	123	42	45	24	14	17	142
18	F	36	38	55	20	13	9	135	45	58	21	14	12	150	50	61	26	18	18	173
19	F	48	22	24	20	0	0	66	28	33	22	0	0	83	39	48	27	0	0	114
20	M	108	38	25	25	0	0	88	41	32	27	5	11	116	49	43	30	5	18	145
21	M	19	35	26	19	0	0	80	39	31	21	1	0	92	44	40	24	1	0	109
22	M	72	25	28	18	0	0	71	29	35	20	0	0	84	33	41	23	0	0	97
23	M	48	37	27	23	0	0	87	41	36	24	0	0	101	48	43	16	1	0	108

24	M	48	35	24	20	0	0	79	39	30	23	0	0	92	49	35	28	0	0	112
25	M	36	36	52	29	30	47	194	45	59	31	32	47	214	48	62	35	33	48	226
26	F	96	40	31	18	1	0	90	43	39	19	1	0	102	48	49	21	1	0	119
27	M	18	37	34	24	0	15	110	43	38	29	26	24	160	49	45	30	28	26	178

Supplementary table 2. FMFM scores of patients accepted double-course hWJSCs transplantation

			Baseline						6-month after treatment						12-month after treatment					
NO.	gender	age	A	B	C	D	E	Total	A	B	C	D	E	Total	A	B	C	D	E	Total
1	F	36	9	15	3	3	1	31	14	24	13	6	3	60	17	32	17	10	3	79
2	F	36	8	18	14	14	13	67	13	24	20	19	19	95	19	29	23	24	19	114
3	F	7	10	4	0	0	0	14	13	17	8	0	0	38	17	19	15	4	0	55
4	M	24	11	16	10	12	6	55	16	25	16	14	10	81	19	29	22	19	10	99
5	M	36	13	13	9	3	5	43	14	18	15	7	5	59	19	25	19	13	9	85
6	F	96	11	23	13	16	28	91	13	23	22	22	35	115	17	29	24	29	36	135
7	F	36	13	1	0	0	0	14	16	16	5	2	0	39	18	20	13	7	0	58
8	F	10	13	0	0	0	0	13	15	7	4	4	0	30	17	13	12	9	0	51
9	M	60	14	22	16	13	24	89	16	26	23	23	28	116	18	29	24	27	30	128
10	F	24	7	4	0	0	0	11	15	9	5	3	0	32	17	18	14	8	0	57
11	M	72	6	19	14	9	16	64	10	23	20	17	19	89	13	27	24	24	20	108
12	M	48	8	12	7	2	0	29	14	17	11	3	0	45	17	22	18	9	0	66
13	M	96	11	24	15	10	25	85	17	27	22	27	32	125	18	32	24	29	33	136
14	F	36	13	18	16	16	13	76	16	22	20	19	16	93	19	29	24	24	16	112
15	M	24	17	15	13	4	8	57	17	19	17	9	10	72	20	27	23	14	10	94
16	M	36	11	20	11	6	14	62	14	25	16	12	16	83	17	30	22	15	17	101
17	M	132	9	14	11	5	6	45	12	17	15	5	6	55	13	17	19	15	6	70
18	F	36	11	22	17	18	17	85	12	27	22	23	22	106	15	30	24	30	28	127

19	F	48	12	1	0	0	0	13	14	7	1	1	0	23	18	15	9	5	0	47
20	M	108	9	16	13	13	1	52	12	19	16	13	1	61	15	21	19	19	4	78
21	M	19	6	3	0	1	0	10	10	10	2	1	0	23	13	11	7	9	0	40
22	M	72	11	1	0	0	0	12	13	5	1	0	0	19	15	10	5	10	0	40
23	M	48	12	1	6	3	2	24	15	5	12	3	2	37	17	9	13	7	5	51
24	M	48	13	8	3	1	1	26	16	14	5	1	1	37	17	15	12	4	6	54
25	M	36	15	22	20	20	25	102	16	27	23	26	30	122	18	30	23	28	31	130
26	F	96	8	22	18	15	15	78	10	26	22	18	19	95	14	30	23	20	19	106
27	M	18	15	12	8	2	1	38	18	17	11	7	1	54	18	17	13	14	1	63

Supplementary table 3. GMFM scores of patients accepted one-course hWJSCs transplantation

			Baseline						6-month after treatment					
NO.	gender	age	A	B	C	D	E	Total	A	B	C	D	E	Total
1	M	84	40	15	2	2	0	59	41	23	2	3	0	69
2	M	72	35	3	0	0	0	38	35	6	0	0	0	41
3	M	84	51	59	40	30	44	224	51	60	40	31	48	230
4	M	108	49	49	25	5	8	136	49	54	25	5	8	141
5	M	84	8	4	0	0	0	12	8	4	0	0	0	12
6	M	96	49	52	23	2	6	132	50	56	23	3	6	137
7	M	108	51	56	31	33	50	221	51	59	31	33	50	224
8	F	120	26	11	0	0	0	37	26	14	0	0	0	40
9	F	108	48	9	6	2	2	67	48	17	6	2	2	75
10	M	96	51	58	42	37	68	256	51	59	42	38	69	259
11	F	84	4	7	0	0	0	11	8	7	0	0	0	15
12	M	96	49	58	37	31	44	199	51	58	37	31	44	201
13	F	108	21	23	3	0	0	47	27	26	3	0	0	56

14	M	72	18	1	0	0	0	19	20	6	0	0	0	26
15	M	84	48	58	38	32	55	231	48	58	38	32	55	231
16	F	132	49	59	34	26	21	189	49	59	34	26	21	189
17	F	72	28	8	0	0	0	36	28	8	0	0	0	36
18	F	84	45	31	3	1	5	85	47	32	4	2	5	90
19	M	108	46	35	16	1	0	98	48	35	16	1	0	100
20	F	132	51	60	40	35	64	250	51	60	40	35	64	250
21	F	72	0	0	0	0	0	0	4	2	0	0	0	6
22	M	72	27	18	4	0	0	49	33	20	4	0	0	57
23	M	144	51	60	38	35	49	233	51	60	38	35	49	233
24	F	72	40	44	20	29	15	148	42	44	20	29	16	151
25	M	96	6	5	0	0	0	11	9	5	0	0	0	14
26	M	121	50	60	42	31	48	231	51	60	42	31	48	232
27	M	72	13	9	0	0	0	22	13	15	0	0	0	28
28	M	144	36	40	1	18	20	115	36	40	1	18	20	115
29	F	120	47	57	33	20	12	169	47	57	34	30	14	182
30	M	82	51	60	42	35	68	256	51	60	42	35	68	256

Supplementary table 4. FMFM scores of patients accepted one-course hWJSCs transplantation

			Baseline						6-month after treatment					
NO.	gender	age	A	B	C	D	E	Total	A	B	C	D	E	Total
1	M	84	21	10	0	4	3	38	21	13	0	7	3	44
2	M	72	1	13	8	3	1	26	1	13	8	3	1	26
3	M	84	21	23	23	29	30	126	21	24	23	29	31	128
4	M	108	21	24	24	30	35	134	21	24	24	30	35	134
5	M	84	10	0	0	0	0	10	14	0	0	0	0	14

6	M	96	9	21	23	23	23	99	9	21	23	24	23	100
7	M	108	21	19	18	23	26	107	21	22	19	23	26	111
8	F	120	21	1	0	0	0	22	21	1	0	0	0	22
9	F	108	21	22	24	22	26	115	21	22	24	22	26	115
10	M	96	21	23	24	29	30	127	21	23	24	29	30	127
11	F	84	10	1	0	0	0	11	10	7	0	0	0	17
12	M	96	21	23	24	28	33	129	21	23	24	28	33	129
13	F	108	14	2	0	0	0	16	14	4	0	0	0	18
14	M	72	2	0	0	0	0	2	6	4	1	0	0	11
15	M	84	21	22	24	25	34	126	21	22	24	25	34	126
16	F	132	21	23	24	27	36	131	21	23	24	27	36	131
17	F	72	17	7	8	1	0	33	17	7	8	1	0	33
18	F	84	21	23	21	21	25	111	21	23	21	21	25	111
19	M	108	21	22	23	27	28	121	21	22	23	27	28	121
20	F	132	21	24	24	29	36	134	21	24	24	29	36	134
21	F	72	2	0	0	0	0	2	4	0	0	0	0	4
22	M	72	8	10	11	3	2	34	8	10	11	3	2	34
23	M	144	21	24	24	29	34	132	21	24	24	29	34	132
24	F	72	8	18	22	21	19	88	8	18	22	21	19	88
25	M	96	21	1	0	0	0	22	21	1	0	0	0	22
26	M	121	12	16	14	20	12	74	12	16	14	20	12	74
27	M	72	17	2	0	0	0	19	17	2	0	0	0	19
28	M	144	16	2	0	0	0	18	16	2	0	0	0	18
29	F	120	19	24	24	30	35	132	19	24	24	30	35	132
30	M	82	21	24	24	29	36	134	21	24	24	29	36	134
